# IgM deposition is a risk factor for delayed remission and early relapse of the pediatric minimal change disease

**DOI:** 10.3389/fped.2023.1072969

**Published:** 2023-02-06

**Authors:** Tao Ju, Yingchao Peng, Yaqin Wei, Xiaojie Li, Meiqiu Wang, Ren Wang, Xiao Yang, Zhiqiang Zhang, Chunlin Gao, Zhengkun Xia

**Affiliations:** ^1^ Department of Pediatrics, Jinling Hospital, Nanjing Medical University, Nanjing, China; ^2^ Affiliated Jinling Hospital, Medical School of Nanjing University, Nanjing, China; ^3^ Department of Pediatrics, Jinling Hospital, The First School of Clinical Medicine, Southern Medical University, Nanjing, China; ^4^ Department of Pediatrics, Jinling Hospital, Nanjing, China

**Keywords:** IgM deposition, minimal change diease, children, remission, relapse

## Abstract

**Background:**

Minimal change disease (MCD) is the most common pathological subtype of pediatric idiopathic nephrotic syndrome (INS). It has been suggested that IgM deposition might predict kidney function deterioration in the course of MCD. However, the specific role of IgM deposition in the prognosis of MCD is still controversial. This study aims to investigate the clinical significance of IgM deposition on delayed remission and early relapse in a pediatric population.

**Methods:**

This study enrolled 283 children diagnosed with MCD by renal biopsy in a single center from 2010 to 2022. These cases were divided into two groups according to the histopathological deposition of IgM. Patients' demographics, clinical parameters, and follow-up data were collected and analyzed. The primary and secondary outcomes were defined as the time to the first remission and the first relapse.

**Results:**

The IgM-positive group had a weaker response to steroids (steroid-sensitive: 23.5% vs. 40.8%; steroid-dependent: 74.0% vs. 51.0%; steroid-resistant: 18.4% vs. 8.2%, *P* = 0.001), and showed more recurrent cases (47.2% vs. 34.4%, *P* = 0.047) compared with the IgM-negative group. The Kaplan-Meier analysis showed that the IgM-positive group had a lower cumulative rate of the first remission (Log-rank, *P* < 0.001) and a higher rate of the first relapse (Log-rank, *P* = 0.034) than the IgM-negative group. Multivariate Cox analysis showed that IgM deposition was independently associated with the delayed first remission (hazard ratio [HR] = 0.604, 95% confidence interval [CI] = 0.465–0.785, *P* < 0.001) and the early first relapse (HR = 1.593, 95% CI = 1.033–2.456, *P* = 0.035).

**Conclusion:**

IgM deposition was associated with a weaker steroid response. MCD children with IgM deposition were prone to delayed first remission and early first relapse.

## Introduction

Primary glomerular disease in children commonly presents as nephrotic syndrome (NS), characterized by massive proteinuria, hypoproteinemia, and peripheral edema. NS includes a variety of pathological subtypes, of which MCD accounts for about 70%–90% of children >1 year of age (1). Most children achieve complete remission (CR) after receiving a course of steroid therapy but are prone to relapse. About 40%–50% of children with MCD, which may progress to frequent relapses or are steroid dependent, require immunosuppressive agents treatment other than steroid (2). However, this proportion varies between different regions and countries.

The role of IgM deposition in the progression and prognosis of MCD is controversial. A report in 2019 showed that IgM deposition was associated with hypertension, steroid dependence, and Chronic kidney disease (CKD) in children with MCD, which may be a predictor of deterioration of renal function (3). Sandra et al. believed that IgM deposition did not affect the outcome of children with MCD (4). In an adult-set cohort study, IgM deposition had a high risk of relapses (5). But the relevant studies on the correlation between IgM deposition and the prognosis of children with MCD needed to be included. In this cohort study, we explored the clinical significance of IgM deposition in MCD by assessing demographic characteristics and clinical features at biopsy, the time to the first remission and the first relapse.

## Methods

### Patients

In this retrospective study, children (≤18 years) with MCD diagnosed by kidney biopsy from 2010 to 2022 were recruited from Jinling hospital. We enrolled cases who met the following criteria: (1) 24-h urine protein to creatinine ratio (UP/CR) ≥0.2 g/g; (2) patients with MCD whose biopsies were performed at any age between 1 and 18 years. Individuals with one of the following conditions were excluded: (1) infectious diseases (e.g., hepatitis B or C, tuberculosis, AIDS); (2) individuals of contemporary autoimmune disease, diabetes mellitus, malignant tumors; (3) drug-induced NS; (4) missing data on IgM deposition in renal pathology; (5) the glomerular number on light microscopy <10.

### Clinicopathologic data and definition

All clinical and pathological data were obtained retrospectively from medical records. The baseline investigations were taken during the kidney biopsy. Follow-up data, including treatment, steroid response, time to the first remission and relapse, was collected and analyzed at 1, 2, 3, 6, 12 months and longer follow-up. The last follow-up time was estimated from the biopsy to the previous encounter in our center. The diagnosis of hypertension was based on the 2017 AAP Blood Pressure Clinical Practice Guidelines (6). The estimated glomerular filtration rate (eGFR) was calculated using the Schwartz formula for children aged 16 years or less (7), and the CKD-EPI recipe for children aged 17 years or older (8).

(1) IgM-positive was defined as the intensity of the IgM staining by immunofluorescence ≥1+. (2) Grade of tubulointerstitial injury scored as none (<10%), mild (10%–24%), moderate (25%–49%), and severe (≥50%), with the percentages mentioned in parentheses. The change of chronic tubulointerstitial injury (CTI) indicated tubular atrophy/interstitial fibrosis. The manifestation of acute tubulointerstitial injury (ATI) was interstitial edema, inflammatory cellular infiltration, and necrosis in tubular epithelia. (3) Steroid-sensitive was defined as CR after the 4 weeks of full-dose oral corticosteroid (equivalent to a prednisone dose of 60 mg/m^2^/day or 2 mg/kg/day) and no relapse during corticosteroid therapy. (4) Steroid-resistant was defined as a lack of CR for at least 4 weeks of full-dose oral prednisone. (5) Steroid-dependent was defined as follows: (1) Reach CR after the 4 weeks of full-dose oral corticosteroid; (2) Two consecutive relapses during corticosteroid therapy or within 15 days of discontinuing corticosteroid therapy; (3) Infectious factors should be excluded at the same time; (4) When relapse occurs, patients could reach CR once the corticosteroid dose is increased (either at full dose or beyond the relapse dose).

### Outcomes definition

Partial remission (PR) was a decrease in 24-h UP/Cr to <2 g/g but >0.2 g/g (or >20 and <200 mg/mmol) with a 50% reduction from its peak value, or the test result of the dipstick decreases by at least 1+, but it does not reach negative. CR was a decrease in 24-h UP/Cr ≤0.2 g/g (or 20 mg/mmol or negative or trace dipstick) on three or more consecutive occasions. Remission was the achievement of PR or CR. Relapse was defined as an increase in UP/Cr to ≥2.0 g/g in cases with a 50% increase from its valley value or in urine dipstick to ≥3+after the remission. Frequent relapse was defined as ≥2 relapses per 6 months or ≥4 relapses per 12 months.

This cohort study's primary and secondary endpoints were the first remission and the first relapse, respectively. When patients achieved the first remission after biopsy, we considered this the first remission. And the time interval from treatment to the first day of remission would be recorded. Time to the first relapse was the time interval from the first remission to the occurrence of the first relapse.

### Statistical analysis

The SPSS 26.0, Graph Pad Prism 8.0, and Adobe Illustrator 2020 were used for statistical analysis in this cohort study. The continuous variables were compared between groups using the Mann-Whitney *U* test, represented by the median (lower quartile, upper quartile), while categorical variables using the chi-square test or Fisher exact test were expressed as frequency (percentage). All data were tested two-sided, with *P* < 0.05 regarded as statistically significant.

## Results

### Baseline demographic and clinical features

**Figure 1:**
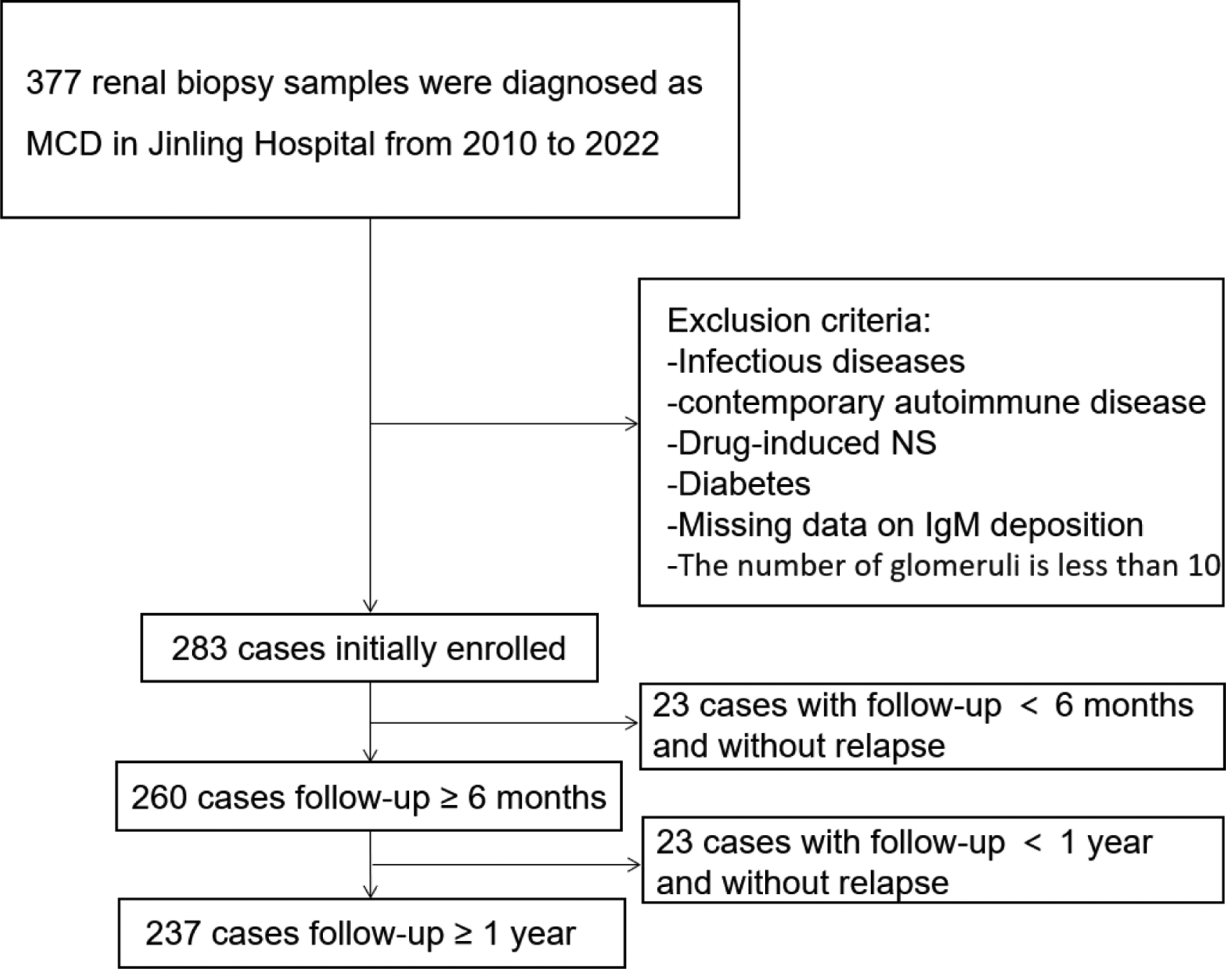
The flowchart shows 283 enrolled patients, 260 with a follow-up of more than 6 months, and 237 with a follow-up of more than 1 year.

As shown in Figure 1, from 2010 to 2022, 283 children were diagnosed with MCD based on renal biopsy in our single center, including 136 (48.1%) in the IgM-positive group and 147 (51.9%) in the IgM-negative group. Baseline demographic characteristics and clinical features at renal biopsies of these children are listed in Table 1. Compared the two groups, there were no statistically significant differences in age of onset, gender, BMI, hypertension, duration, serum IgG, serum IgM, serum C3, proteinuria levels, hematuria cases, Acute Kidney Injury (AKI), total glomeruli, ATI and CTI (All *P *> 0.05). No severe ATI and moderate to severe CTI were observed in the renal pathology of all MCD individuals.

**Table 1:** Demographic characteristics and clinical features at biopsy.

Items	MCD 283 (100%)	IgM+ 136 (48.1%)	IgM− 147 (51.9%)	*P*
**Male, *n* (%)**	217 (76.7)	104 (76.5)	113 (76.9)	1.000
**Age of onset, years**	12.0 (5.5–15.7)	10.3 (4.0–16.7)	13.8 (7.4–16.3)	0.063
**Age at biopsy, years**	14.2 (10.2–16.3)	12.6 (6.1–15.8)	15.2 (12.4–16.9)	**<0.001**
**BMI, kg/m^2^**	20.5 (18.2–23.0)	20.4 (18.0–22.8)	20.5 (18.4–23.4)	0.272
**Hypertension, *n* (%)**	103 (36.4)	53 (39)	50 (34)	0.39
**Duration, months**	3 (1–26)	3 (1–16)	2 (1–32)	0.768
**Serum albumin, g/L**	22.8 (18.6–30.1)	22 (17.7–27.9)	24 (19.7–31.5)	**0.02**
**Serum IgG**	2.9 (2.0–5.0)	2.7 (1.6–4.2)	3.3 (2.5–5.6)	0.13
**Serum IgM**	1.4 (1.1–1.9)	1.4 (1.1–1.8)	1.5 (1.1–2.0)	0.30
**Serum C3**	1.2 (1.0–1.4)	1.1 (1.0–1.3)	1.2 (1.0–1.4)	0.31
**Proteinuria, g/day**	3.48 (1.4–6.7)	3.62 (1.44–7.79)	3.22 (1.36–6.33)	0.153
**Hematuria, *n* (%)**	106 (37.5)	55 (40.4)	51 (34.7)	0.328
**eGFR, ml/min/1.73 m^2^**	160 (135.6–202.8)	171.9 (140.0–213.8)	148.9 (134.1–191.0)	**0.002**
**AKI, *n* (%)**	8 (2.8)	3 (2.2)	5 (3.4)	0.724
**Total glomeruli**	24 (17–31)	24 (18–31)	25 (17–31)	0.597
**ATI**				
None, *n* (%)	28 (9.9)	10 (7.4)	18 (12.2)	0.145
Mild, *n* (%)	253 (89.4)	126 (92.6)	127 (86.4)	
Moderate, *n* (%)	2 (0.7)	0 (0)	2 (1.4)	
Severe, *n* (%)	0 (0)	0 (0)	0 (0)	
**CTI**				
None, *n* (%)	251 (88.7)	121 (89.0)	130 (88.4)	1.000
Mild, *n* (%)	32 (11.3)	15 (11.0)	17 (11.6)	
Moderate, *n* (%)	0 (0)	0 (0)	0 (0)	
Severe, *n* (%)	0 (0)	0 (0)	0 (0)	
**C3 deposition, *n* (%)**	23 (8.1)	16 (11.8)	7 (4.8)	**0.048**

Values are median (IQR) unless stated otherwise; *P* < 0.05 vs. IgM-negative group; BMI, body mass index; eGFR, estimated glomerular filtration rate; AKI, acute kidney injury; Duration, the interval time between the onset and the biopsy; ATI, acute tubulointerstitial injury; CTI, chronic tubulointerstitial injury.

*P* values less than 0.05 are highlighted in bold.

The median age at the renal biopsies of patients was 12.6 years old for the IgM-positive group and 15.2 years for the IgM-negative group (*P* < 0.001). In immunofluorescence, 16 (11.8%) patients also had positive C3 deposition in the IgM-positive group, while only 7 (4.8%) were in the IgM-negative group (*P* = 0.048). The IgM-positive group had a lower level of serum albumin (median, IQR: 22, 17.7–27.9 g/L vs. 24, 19.7–31.5 g/L, *P* = 0.02) and a higher level of eGFR (median, IQR: 171.9, 140.0–213.8 ml/min/1.73 m^2^ vs. 148.9, 134.1–191.0 ml/min/1.73 m^2^, *P* = 0.002) than the IgM-negative group.

### Treatment and response

A summary of treatment and outcomes is shown in Table 2. The median follow-up duration is 30.5 months for the IgM-positive group and 49 months for the IgM-negative group. Steroid therapy was administered to 283 patients, 42 of whom were treated with angiotensin-converting enzyme inhibitors (ACEI). 210 patients received immunosuppressive agents other than steroid therapy, which included 107 (78.7%) cases for the IgM-positive group and 103 (70.1%) for the IgM-negative group. There were no statistically significant differences in the data collected above. But the IgM-positive group had a significantly higher proportion of patients who received rituximab (22.6% vs. 11.6%, *P* = 0.025) than the IgM-negative group. More details of immunosuppressive therapy for the steroid-sensitive nephrotic syndrome (SSNS), steroid-dependent nephrotic syndrome (SDNS) and steroid-resistant nephrotic syndrome (SRNS) patients are shown in Supplementary Tables S1–S4.

**Table 2:** Treatment and outcomes of minimal change disease.

Items	MCD 283 (100%)	IgM+ 136 (48.1%)	IgM− 147 (51.9%)	*P*
**Follow-up, months**	35 (17–59)	30.5 (13–42.8)	49 (20–76)	0.114
**CKD, *n* (%)**	0 (0)	0 (0)	0 (0)	
**Treatment, *n* (%)**				
ACEI/ARB	42 (14.8)	15 (11)	27 (18.4)	0.095
Steroid	283 (100)	136 (100)	147 (100)	
**Immunosuppressive agents other than steroid, *n* (%)**	210 (74.2)	107 (78.7)	103 (70.1)	0.105
**Rituximab, *n* (%)**	46 (17.7)	30 (22.6)	17 (11.6)	**0.025**
**Steroid response, *n* (%)**				
Steroid-sensitive	92 (32.5)	32 (23.5)	60 (40.8)	**0.001**
Steroid-dependent	154 (54.4)	79 (74.0)	75 (51.0)	
Steroid-resistant	37 (13.1)	25 (18.4)	12 (8.2)	
**Treatment response**				
First remission, days	30 (17.8–41)	33 (19–50)	27 (15–37)	**0.004**
**Relapse, *n* (%)**				
None	111 (39.2)	46 (33.8)	65 (44.2)	0.182
Frequent relapses	57 (20.1)	28 (20.6)	29 (19.7)	
Infrequent relapses	114 (40.3)	61 (44.9)	53 (36.1)	
**Remission at the end follow-up, *n* (%)**				
None	37 (13.1)	21 (15.4)	16 (10.9)	0.147
Complete remission	61 (21.6)	23 (16.9)	38 (25.9)	
Partial remission	185 (65.4)	92 (67.6)	93 (63.3)	
Remission	246 (86.9)	115 (84.6)	131 (89.1)	0.292

CKD, chronic kidney disease (eGFR less than 60 ml/min/1.73 m^2^); ACEI, angiotensin-converting enzyme inhibitors; ARB, angiotensin receptor blockers.

*P* values less than 0.05 are highlighted in bold.

The IgM-positive group was less responsive to steroid therapy than the IgM-negative group (*P* = 0.001). For instance, the IgM-positive group has fewer steroid-sensitive patients (23.5% vs. 40.8%), more steroid-dependent (74% vs. 51%) and steroid-resistant patients (18.4% vs. 8.2%) than the IgM-negative group. 283 (100%) patients reached the first remission during a median follow-up time of 30 days. The median time to the first remission was significantly different between the two groups (median, IQR; IgM-negative group = 27, 15–37 days vs. IgM-positive group = 33, 19–50 days, *P* = 0.004). 171 (60.8%) patients relapsed at least once during a median follow-up time of 200 days. Although the frequencies of frequent relapses (20.6% vs. 19.7%) and infrequent relapses (44.9% vs. 36.1%) were more common in the IgM-positive group than in the IgM-negative group, there was no statistically significant difference in relapse between the two groups (*P* = 0.182).

### Predictors of the first remission and relapse

In total, 260 cases were followed up for more than 6 months. And we included these patients to detect the predictors of the first remission and relapse.

**Figure 2:**
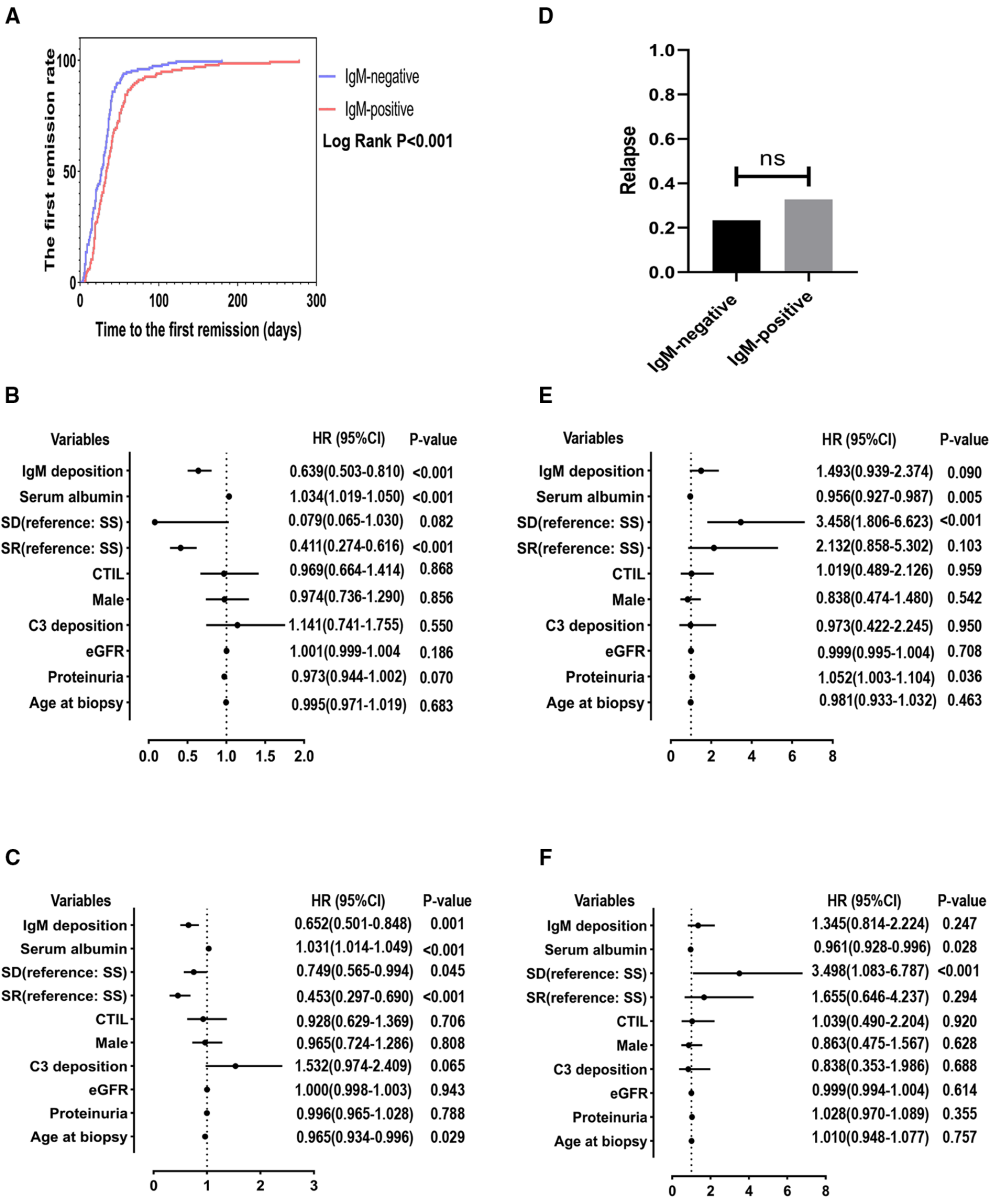
The minimum follow-up was 6 months. SD, steroid-dependent; SR, steroid-resistant; SS, steroid-sensitive; eGFR, estimated glomerular filtration rate; CTI, chronic tubulointerstitial injury. (**A**) Cumulative rate of the first remission. (**B**) Independent predictors of the first remission by univariate cox regression analysis. (**C**) Independent predictors of the first remission by multivariate cox regression analysis. (**D**) Comparison of the number of recurrences between the two groups. (**E**) Independent predictors of the first relapse by univariate cox regression analysis. (**F**) Independent predictors of the first relapse by multivariate cox regression analysis.

The Kaplan-Meier survival analysis showed that the IgM-positive group had a lower cumulative rate of the first remission than the IgM-negative group (Log-rank, *P* < 0.001) (Figure 2A). The 1-, 2-, 3- and 6-month remission rates were 57.14% and 43.42%, 94.56% and 85.86%, 96.60% and 92.56%, 100.00% and 98.51% for the IgM-negative group and the IgM-positive group, respectively. The result of the univariate Cox regression analysis is shown in Figure 2B. IgM deposition (hazard ratio [HR] = 0.639, 95% confidence interval [CI] = 0.503–0.810, *P* < 0.001), serum albumin (HR = 1.034, 95% CI = 1.019–1.050, *P* < 0.001), steroid-resistant (Referance = steroid-sensitive; HR = 0.411, 95% CI = 0.274–0.616, *P* < 0.001) were associated with the first remission. The result of the multivariate Cox regression analysis is listed in Figure 2C. IgM deposition (HR = 0.625, 95% CI = 0.501–0.848, *P* = 0.001), serum albumin (HR = 1.031, 95% CI = 1.014–1.049, *P* < 0.001), steroid-dependent (Referance = steroid-sensitive; HR = 0.749, 95% CI = 0.565–0.994, *P* = 0.045) and steroid-resistant (Referance = steroid-sensitive; HR = 0.453, 95% CI = 0.297–0.690, *P* < 0.001) were independently association with the first remission.

However, there is no statistical difference in relapses between the positive and negative groups (IgM-negative group = 23.4% vs. IgM-positive group = 32.8%, *P *> 0.05) (Figure 2D). Moreover, as shown in the Figures 2E, F, the univariate (HR = 1.493, 95% CI = 0.939–2.374, *P* = 0.090) and multivariate analysis (HR = 1.345, 95% CI = 0.814–2.224, *P* = 0.247) showed that IgM deposition was not associated with the first relapse. Serum albumin (HR = 0.956, 95% CI = 0.927–0.987, *P* = 0.005) and steroid-dependent (Referance = steroid-sensitive; HR = 3.458, 95% CI = 1.806–6.623, *P* < 0.001) were independently associated with the first relapse in the univariate Cox analysis. In the multivariate Cox analysis, serum albumin (HR = 0.961, 95% CI = 0.928–0.996, *P* = 0.028) and steroid-dependent (Referance = steroid-sensitive; HR = 3.498, 95% CI = 1.083–6.787, *P* < 0.001) were still independently associated with the first relapse.

**Figure 3:**
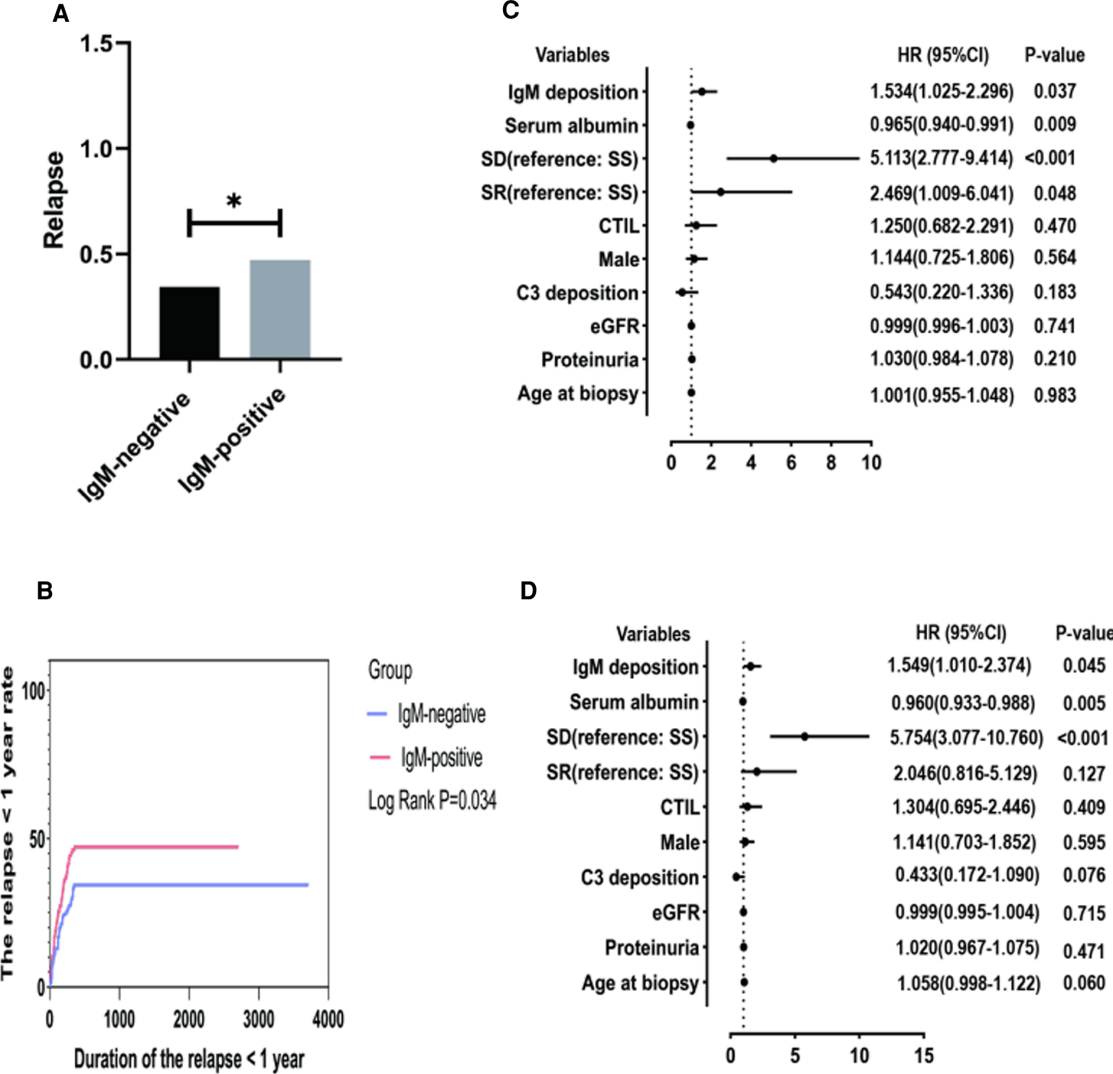
The minimum follow-up was 1 year. SD, steroid-dependent; SR, steroid-resistant; SS, steroid-sensitive; eGFR, estimated glomerular filtration rate; CTI, chronic tubulointerstitial injury; (**A**) Comparison of relapses between the two groups. (**B**) Cumulative rate of the first remission. (**C**) Independent predictors of the first relapse by univariate cox regression analysis. (**D**) Independent predictors of the first relapse by multivariate cox regression analysis.

We suggested that a significant portion of patients relapsed after a follow-up of 1 year. Therefore, extended follow-up may be necessary to demonstrate the significance of IgM deposition in relapse. Finally, 237 patients with a follow-up of more than 1 year were included to detect the predictors of the first relapse. The results are shown in Figure 3. Figure 3A shows that the IgM-positive group had a significantly higher proportion of patients relapse (47.2% vs. 34.4%, *P* = 0.047). Figure 3B shows that the IgM-positive group has a significantly higher cumulative rate of the first relapse (Log-rank, *P* = 0.034). The 3-month, 6-month, 1-year and 2-year remission rates were 12.98% and 18.87%, 21.37% and 30.19%, 34.35% and 41.17%, 34.35% and 47.17% for the IgM-negative group and the IgM-positive group, respectively. The univariate Cox regression analysis showed IgM deposition (HR = 1.534, 95% CI = 1.025–2.296, *P* = 0.037) and serum albumin (HR = 0.965, 95% CI = 0.940–0.991, *P* = 0.009) were associated with the first relapse. Figure 3D shows that IgM deposition (HR = 1.549, 95% CI = 1.010–2.374, *P* = 0.045) and serum albumin (HR = 0.965, 95% CI = 0.940–0.991, *P* = 0.009) are independently associated with the first relapse.

## Discussion

Several studies have examined the clinical characteristics and renal outcomes in pediatric MCD with or without IgM deposition (3, 9, 10). However, the role of glomerular IgM depositions remains controversial. Moreover, a small sample size limited most observational studies among children. In this sizeable consecutive retrospective study, we analyzed the clinicopathologic characteristics, steroid response, remission and relapse in pediatric MCD with and without IgM deposition. Our patients with IgM deposition showed a weaker steroid response. The multivariate regression analysis revealed that IgM deposition was associated with delayed remission and early relapse. Currently, this study is the largest one to detect the clinical significance of IgM deposition in children with MCD.

In previous studies, MCD children with IgM deposition showed a more common frequency of steroid dependence (3, 11). A study analyzed the data of 55 children with steroid-resistant or steroid-dependent MCD and found that 23 (41.82%) patients had IgM deposition (12). Our analysis yielded similar results, which revealed that patients with IgM deposition showed a higher frequency of steroid resistance and steroid dependence. In addition, we found that children with IgM-positive deposition had lower serum albumin levels, suggesting that this may be associated with a more severe disease state. Conversely, one pediatric study and one adult study found no difference between MCD patients with or without IgM deposition (5, 9). Regardless of histopathological subtype, patients with mesangial IgM deposition had a weaker response to steroids (13), but another study proved otherwise (14). It should be noted that although there was a statistical difference in the baseline level of eGFR between the two groups, eGFR was not significant in COX regression analysis for the endpoints. In addition, the baseline level of eGFR between the two groups was normal. Moreover, no patients developed CKD during the follow-up period of this study. So we consider this statistical difference to be of no practical clinical significance.

In our study, the time to the first remission was significantly longer in the IgM-positive group than in the IgM-negative group. And the IgM deposition was an independent risk factor for delayed remission. Nevertheless, Mateja et al. found no differences in the time to the first remission and frequency of the first remission between MCD children with and without IgM deposition (9). An adult study yielded similar results (5). The findings on remission are controversial, and further studies among children need to be performed.

A study published in 2021 indicated that IgM mesangial deposition was a risk factor for relapses of adult-onset MCD (5). In our study, the Cox regression analysis showed no signs of IgM deposition in relapse when a minimum follow-up was set to 6 months. Intriguingly, IgM deposition became an independent predictor of relapse when a minimum follow-up was extended to 1 year. The discrepant results might come from different minimum follow-up times. The Cox regression model, the most popular survival regression model, investigates the relationship between predictors and the time-to-event (15). After the first remission, about 70% of children patients suffer relapses for 18–24 months (1), indicating that most relapse events occur 1.5 years after the first remission. Our study showed a similar frequency of relapse, and the median time to relapse after the first remission was 200 days. We suggested a minimum follow-up of 6 months was too short to observe enough relapse events.

IgM deposition can be observed in some MCD and focal segmental glomerulosclerosis (FSGS) patients' renal biopsy samples under immunofluorescence (16, 17). MCD and idiopathic FSGS have the same dominant pathogenesis, podocyte injury. Maas et al. put forward that MCD and idiopathic FSGS are different manifestations of the same progressive disease (18). In fact, not all MCD cases will progress to FSGS. A report in 2001 showed that 64 children with positive IgM deposition were more likely to progress to FSGS or renal function during long-term follow-up (10). Many researchers have proposed the IgM nephropathy (IgMN) concept and believe that IgMN is a transitional state between MCD and FSGS (12, 19, 20). These researches indicated that IgM deposition in MCD might play an essential role in the evolution of FSGS. Because Few patients accepted repeat renal biopsies, our study did not collect and analyze the data of repeat biopsies.

IgM is the largest serum antibody in the human body and the initial activator for the complement cascade (21). Some scholars believed that IgM natural antibodies could bind to the *de novo* antigens exposed to the glomerulus, activate the complement system, and further contribute to renal injury (22, 23). Whether MCD patients may have an activated complement system deserves further investigation. Long-term remission and low recurrence rate are vital to a favorable prognosis of MCD. Therefore, we suggest intensive monitoring of disease activity in MCD children with IgM deposition.

There were some limitations to our study. First, our study still had the inevitable drawbacks of a retrospective cohort design, indicating that the patients needed to accept uniform treatment. And we did not adjust the immunosuppressive therapy in the Cox regression model. However, it should be noted that there was no difference between patients with and without IgM deposition using ACEI/ARB or steroid or immunosuppressive agents, as shown in Table 2. Second, not all patients were examined by electron microscope for the degree of foot process fusion, so it was impossible to compare the degree of foot process fusion between groups. Third, we did not adopt the Definition of IgMN, for its pathophysiology is controversial. Fourth, Our cohort study only looked at short-term outcomes, and long-term outcomes are not known at this time, so more prospective studies are needed before considering IgM-positive deposition as an adverse prognostic factor for pediatric MCD in clinical practice.

## Conclusion

MCD children with IgM deposition are more likely to present with steroid dependence or steroid resistance, suggesting a possible association with a poorer disease state. IgM deposition is an independent risk factor for delayed remission and an independent promoting factor for early relapse of pediatric MCD. Therefore, enhanced disease activity surveillance should be considered in MCD pediatric patients with IgM deposition. Nonetheless, a prospective study is needed to verify the function of IgM deposition in MCD outcomes.

## Data Availability

The raw data supporting the conclusions of this article will be made available by the authors, without undue reservation.
