# Supplementary material for: IgM deposition is a risk factor for delayed remission and early relapse of the pediatric minimal change disease
Source: Front Pediatr. 2023 Feb 6;11:1072969. doi: 10.3389/fped.2023.1072969 (PMC9936809; doi:10.3389/fped.2023.1072969)
Supplement: Supplementary file 1 [file Datasheet1.pdf]

**Supplementary Table 1**: The use of immunosuppressive agents in steroid-sensitive group (92 cases)

| | TAC | CsA | MMF | LEF | TG | CTX | RTX |
|---|---|---|---|---|---|---|---|
| **Groups, n(%)** | | | | | | | |
| IgM-positive 32 (34.8%) | / | / | / | 8 (25%) | 2 (6.3%) | / | 3 (9.4%) |
| IgM-negative 60 (65.2%) | / | / | / | 22 (36.7%) | 6 (10%) | 2 (3.3%) | / |
| **Remission, n(%)** | | | | | | | |
| IgM-positive | / | / | / | 8 (100%) | 2 (100%) | / | 3 (100%) |
| IgM-negative | / | / | / | 22 (100%) | 1 (16.7%) | 0 (0) | / |

/ None of the patients took this immunosuppressant; *TAC* Tacrolimus; *CsA* Cyclosporin A; *MMF* mycophenolate mofetil; *LEF* Lefunomide;
*TG* Tripterygium glycoside; *CTX* Cyclophosphamide; *RTX* Rituximab

**Supplementary Table 2**: The use of immunosuppressive agents in steroid-dependent group (164 cases)

| | TAC | CsA | MMF | LEF | TG | CTX | RTX |
|---|---|---|---|---|---|---|---|
| **Groups, n(%)** | | | | | | | |
| IgM-positive 79 (51.3%) | 54 (68.4%) | 4 (5.1%) | 2 (2.5%) | 23 (29.1%) | 11 (13.9%) | 5 (6.3%) | 22 (27.8%) |
| IgM-negative 75 (48.7%) | 36 (48%) | 6 (8%) | 3 (4%) | 35 (46.7%) | 17 (22.7%) | 4 (5.3%) | 16 (21.3%) |
| **Remission, n(%)** | | | | | | | |
| IgM-positive | 41 (75.9%) | 1 (25%) | 0 (0) | 22 (95.7%) | 4 (36.4%) | 1 (20%) | 22 (100%) |
| IgM-negative | 30 (83.3%) | 3 (50%) | 3 (100%) | 32 (91.4%) | 9 (52.9%) | 1 (25%) | 13 (81.3%) |

*TAC* Tacrolimus; *CsA* Cyclosporin A; MMF mycophenolate mofetil; *LEF* Lefunomide;
*TG* Tripterygium glycoside; *CTX* Cyclophosphamide; *RTX* Rituximab

**Supplementary Table 3:** The use of immunosuppressive agents in steroid-resistant group (37 cases)

| | TAC | CsA | MMF | LEF | TG | CTX | RTX |
|---|---|---|---|---|---|---|---|
| **Groups, n(%)** | | | | | | | |
| IgM-positive 25 (67.6%) | 15 (60%) | 1 (4%) | / | 3 (12%) | 2 (8%) | / | 4(16%) |
| IgM-negative 12 (32.4%) | 9 (75%) | 1 (8.3%) | / | 6 (50%) | 2 (16.7%) | 2 (16.7%) | 1(8.3%) |
| **Remission, n(%)** | | | | | | | |
| IgM-positive | 14 (93.3%) | 0 (0) | / | 3 (100%) | 0 (0) | / | 4(100%) |
| IgM-negative | 9 (100%) | 0 (0) | / | 6 (100%) | 1 (50%) | 0 (0) | 1(100%) |

/ None of the patients took this immunosuppressant; *TAC* Tacrolimus; *CsA* Cyclosporin A; *MMF* mycophenolate mofetil; *LEF* Lefunomide;
*TG* Tripterygium glycoside; *CTX* Cyclophosphamide; *RTX* Rituximab

**Supplementary Table 4: The use of immunosuppressive agents in IgM group (283 cases)**

| | IgM-positive | | | | IgM-negative | | | |
|---|---|---|---|---|---|---|---|---|
| | SS | SD | SR | Remission | SS | SD | SR | Remission |
| TAC | / | 54 | 15 | 55 (79.7%) | / | 36 | 9 | 39 (86.7%) |
| CsA | / | 4 | 1 | 1 (20%) | / | 6 | 1 | 3 (42.6%) |
| MMF | / | 2 | / | 0 (0) | / | 3 | / | 3 (100%) |
| LEF | 8 | 23 | 3 | 33 (97.1%) | 22 | 35 | 6 | 60 (95.2%) |
| TG | 2 | 11 | 2 | 6 (40%) | 6 | 17 | 2 | 11 (44%) |
| CTX | / | 5 | / | 1 (20%) | 2 | 4 | 2 | 1 (12.5%) |
| RTX | 3 | 22 | 4 | 29 (100%) | / | 16 | 1 | 14 (82.4%) |

/ None of the patients took this immunosuppressant; *TAC* Tacrolimus; *CsA* Cyclosporin A; *MMF* mycophenolate mofetil; *LEF* Lefunomide;
*TG* Tripterygium glycoside; *CTX* Cyclophosphamide; *RTX* Rituximab; *SS* steroid-sensitive; *SD* steroid-dependent; *SR* steroid-resistan
